# Thiol Redox Transitions in Cell Signaling: a Lesson from N-Acetylcysteine

**DOI:** 10.1100/tsw.2010.104

**Published:** 2010-06-29

**Authors:** Tiziana Parasassi, Roberto Brunelli, Graziella Costa, Marco De Spirito, Ewa Krasnowska, Thomas Lundeberg, Eugenia Pittaluga, Fulvio Ursini

**Affiliations:** ^1^ Istituto di Neurobiologia e Medicina Molecolare, CNR, Roma, Italy; ^2^ Dipartimento di Ginecologia e Ostetricia, Università di Roma Sapienza, Roma, Italy; ^3^ Istituto di Fisica, Facoltà di Medicina, Università Cattolica del Sacro Cuore, Roma, Italy; ^4^ FAAB Sabbatsbergs Hospital, Stockholm, Sweden; ^5^ Dipartimento di Chimica Biologica, Università di Padova, Padova, Italy

**Keywords:** antioxidant, c-Src inactivation, differentiation, functional redox switches, gene expression, hydrogen peroxide, junctions, oxidative stress, proliferation, sensitive cysteine

## Abstract

The functional status of cells is under the control of external stimuli affecting the function of critical proteins and eventually gene expression. Signal sensing and transduction by messengers to specific effectors operate by post-translational modification of proteins, among which thiol redox switches play a fundamental role that is just beginning to be understood. The maintenance of the redox status is, indeed, crucial for cellular homeostasis and its dysregulation towards a more oxidized intracellular environment is associated with aberrant proliferation, ultimately related to diseases such as cancer, cardiovascular disease, and diabetes. Redox transitions occur in sensitive cysteine residues of regulatory proteins relevant to signaling, their evolution to metastable disulfides accounting for the functional redox switch. N-acetylcysteine (NAC) is a thiol-containing compound that is able to interfere with redox transitions of thiols and, thus, in principle, able to modulate redox signaling. We here review the redox chemistry of NAC, then screen possible mechanisms to explain the effects observed in NAC-treated normal and cancer cells; such effects involve a modification of global gene expression, thus of functions and morphology, with a leitmotif of a switch from proliferation to terminal differentiation. The regulation of thiol redox transitions in cell signaling is, therefore, proposed as a new tool, holding promise not only for a deeper explanation of mechanisms, but indeed for innovative pharmacological interventions.

## THIOL REDOX CONTROL IN CELL SIGNALING

Redox status is a chemical concept that recently attracted an increasing interest in the field of cell signaling. Indeed, redox switches are seen today as functional post-translational modifications of regulatory proteins, implicated in the control of gene expression and metabolism[1,2,3,4,5].

Several redox couples are involved and connected in redox transitions, with the final outcome of a functional modification of the final effector. We could depict these pathways with an upstream formation of the first oxidant, then sensors and transducers targeting the signal to downstream effectors. Along the way, reductants or, more precisely, redox couples at lower potential counteract or revert the signal brought by oxidants. All this occurs through thermodynamically permitted and kinetically controlled redox transitions.

The condition under which the oxidized form in the different redox couples prevails is usually referred to as "oxidative stress" and is meant to identify an environment prone to oxidation. Among oxygen-derived oxidants, hydrogen peroxide is generated by dismutation of superoxide produced by NADPH oxidase or mitochondrial respiratory chain, and has been shown to be involved in signaling. Other reactive oxygen species have little to do with signaling and metabolic regulations[1].

In proteins, targets of redox signaling by hydrogen peroxide are cysteine residues prone to oxidation. They have been identified as a subproteome of Cys in proteins, a redox-sensitive group opposed to the structural one[2], being metastable and generally dissociated at physiological pH. A lower pKa of the thiol group of a Cys can be due to cationic amino acids in close proximity and is usually seen as the cause of its oxidizability[4,6,7]. However, stricter criteria must be matched in order to warrant the major constraint of kinetic control, in this case of a high rate constant for the oxidation reaction. Indeed, this rate constant has to be very high in order to compete with extremely efficient peroxidases and peroxiredoxins. This competition largely restricts the group of Cys residues prone to oxidation when the concentration of hydrogen peroxide increases.

Upon reaction with the hydroperoxide, specific -S- are transformed into a sulfenic acid residue (-SOH), and if a second Cys is available, into a disulfide (-SS-)[8,9]. Further oxidation is unlikely and generally referred to as a parasite dead-end pathway, not involved in signaling. These peculiar transitions actually define the "functional redox switches" and the formed disulfides generally disobey the canonical rules of protein stereochemistry, being metastable and easier to reduce (higher redox potential) than structural disulfides[2].

Both the control and the reversal of the oxidative pathway are achieved by redox couples at progressively lower redox potential. Together with the oxidant formation, the redox status of reductants is, therefore, the second player in determining the redox status of the environment. Encompassing different couples at different potential, the oxidative potential is thermodynamically driven from hydrogen peroxide to a pyridin nucleotide through a series of intermediates where sulfur undergoes reversible redox switches at different potential. This can be schematically represented by the following series:

Hydrogen peroxide/water; Prot(SS)/Prot$(SH)_2$; GSSG/GSH; Trx(SS)/Trx$(SH)_2$;
Flavoprotein-ox/Flavoprotein-red; NADP/NADPH

where Prot(SS)/Prot$(SH)_2$ are either sensor/transducers of redox signaling or hydroperoxide-reducing enzymes. In this framework, selenoperoxidases can be seen as peculiar cases of redox proteins, where the more reactive selenium substitutes for sulfur.

There is no equilibrium between redox couples, the redox status of different intermediates being kinetically controlled. Indeed, such control requires highly specific enzyme-substrate and protein-protein interactions to permit the redox transitions of Cys residues.

In the framework of these complex pathways, experimental observations associated a shift to a more oxidized redox environment to pathological conditions such as cancer, aberrant wound healing, fibrosis, cardiovascular diseases, diabetes, and neurodegenerative diseases, most of which tightly connected to aberrant proliferation[3].

At cellular level, it is generally accepted that a transiently more oxidant environment primes the progression from G1 to S phase[10], and also generally accepted is the relevance of redox switches of thiols in enzymes, receptors, transporters, transcription factors, structural elements, regulators of protein trafficking synthesis and degradation, and cytoskeletal structure, thus deeply affecting the overall cellular homeostasis[4,11].

The possibility of modulating a cell's behavior or of restoring its homeostasis by changing the redox status is intrinsically appealing in pharmacology and nutrition. Reduced thiols are the first reasonable candidates to accomplish such a function; among them, N-acetylcysteine (NAC) is the most promising.

In the past year, we challenged normal and cancer cells of various origin with NAC, and we described a global rearrangement of gene expression, massive biochemical and morphological changes, all consistent with a shift to terminally differentiated phenotypes and functions[12,13,14,15].

Bearing in mind the most recent discoveries on the relevance of functional switches based on thiol redox transitions and before reviewing the observed effects of NAC, we discuss the possible mechanisms of NAC action, on chemical bases, aiming to figure out common reasonable underlying mechanisms.

## POSSIBLE BASIC REDOX TRANSITIONS INVOLVING NAC

1. NAC could be a free radical scavenger antioxidant. Although popular, this concept is extremely unlikely. NAC, in fact, does not fit the criteria to be a radical scavenger; in the presence of an oxidant-free radical, upon one-electron redox transition, the produced thiyl radical -$S^{\bullet}$ is still enough oxidant to propagate oxidative chain reactions. Moreover, the annihilating radical-radical interaction is limited by concentration of thiyl radicals and thus sulfur is just progressively oxidized[16].
2. NAC could directly reduce hydroperoxides in a nucleophilic displacement reaction. The criticism about this reaction resides in its low rate that renders it quite unlikely in a biological environment – as in the case of GSH[1]. Such a reaction will never compete with much more efficient removal of hydroperoxides by peroxidases, peroxiredoxins, and catalase.
3. NAC could be a substrate for GSH synthesis. Conflicting reports exist in the literature about a substantial GSH increase after NAC supplementation[17,18,19], which would lead to a lower redox potential of the GSSG/GSH couple. Moreover, an increase in the concentration of GSH, although changing the redox potential of the couple, would minimally, if at all, affect the actual concentration of hydrogen peroxide. This indeed depends on the nonreversible reaction of glutathione peroxidases, peroxiredoxins, and the related reductases and, eventually, on the continuous reduction of NADP from glucose oxidation. Thus, the option that an increase in GSH – due to NAC – could substantially affect the steady-state concentration of hydrogen peroxide through an increased activity of GSH peroxidases is extremely unlikely. Such a mechanism, instead, could be relevant only in cases of massive GSH depletion, such as in the peculiar case of the last phase of spermatogenesis[20]. The option of a nonenzymatic reaction of GSH is also already ruled out from the considerations in the previous point 2.
4. NAC could reduce protein disulfides. This is a possible mechanism of NAC action, although it has to compete with the much more efficient enzymatic systems encompassing thioredoxins and glutaredoxins, and the corresponding reductases (TrxR, GR). Nevertheless, also for these interactions, the key role is played by the specificity of protein-protein interactions kinetically driving the reaction. The option that NAC could reduce some specific disulfides sterically inaccessible to thioredoxins and glutaredoxins is therefore still open.
5. NAC could react with sulfenic acid derivatives in proteins. This event is chemically and biologically plausible. Notwithstanding that the "stable" sulfenic acid can only exist in hidden spots of the proteins, usually inaccessible to reducing substrates, the reactivity with NAC is still a reasonable option. Notably, this reaction would be in competition with GSH, but GSH accessibility is expected to be more limited than that of a small molecule such as NAC. When sulfenic acid derivatives are intermediates in the formation of intrachain or mixed disulfides, the reaction with NAC, in competition with the second thiol, would prevent the formation of the disulfide.

In conclusion, from the basic chemistry of redox transitions of NAC, we can conclude that (1) a direct chemical interaction with signaling oxidants (hydroperoxides) is extremely unlikely; (2) the option that NAC acts as precursor of GSH can be relevant only in conditions of extreme GSH depletion; (3) favored over thioredoxins in specific interactions, in some cases NAC can reduce disulfides; (4) by interacting with sulfenic acid derivative in proteins, NAC can interfere with the formation of disulfides involved in functional redox switches and signaling.

These two last options could represent the leitmotif underlying NAC pleiotropic effects upon supplementation to cells. Therefore, more than a generic “antioxidant”, NAC can ultimately be seen as a player interfering with functional redox switches.

## GLOBAL GENE EXPRESSION ANALYSIS OF NAC-TREATED CELLS

In agreement with the concept of an effect in regulation of transcription factors, in cells treated with NAC, we observed an altered pattern of gene expression. Here we report a simplified overview (for a complete analysis, see [12,13]).

Approximately 12,000 previously characterized sequences and a spotted cDNA array comprising approximately 30,000 cDNA clones, with subsequent confirmation by real-time RT-PCR, were explored over time in primary normal human epidermal keratinocytes (NHEK) and in human colon carcinoma cells (Caco-2) treated with NAC at a concentration of 2 or 10 mM, respectively.

In both normal and cancer cells treated with NAC, analysis of differential gene expression and gene ontology indicates a shift from a proliferative to a differentiated profile, with a strong tendency towards inhibition of proliferation and stimulation of differentiation. A relatively limited early response is detected at 1 and 3 h after NAC treatment, followed by an increasingly stronger response at 12 and 24/48 h, also suggesting a long-standing functional shift in cells. Interestingly, the mechanisms inhibiting cell proliferation in NHEK and Caco-2 cell lines appear to differ significantly, being almost completely lineage specific. This is demonstrated by a cluster analysis of gene-specific differences that results in lack of correlation between cell lines, further supported by the gene ontology analysis that indicates distinct mechanisms followed by the two cell lines to achieve their differentiated state.

### Initial Response

A large part of the differentially expressed genes have clear implications in withdrawal of mitogenic signals and in promotion of growth arrest. Multiple signaling pathways are involved, collectively describing molecular changes associated with the mediation of a differentiated epithelial phenotype. Two cases deserve a mention. First, a significant transcriptional down-regulation of the inhibitor of differentiation 1 (ID-1) was found at 1 h after NAC treatment in both Caco-2 and NHEK, suggesting an early common mechanism. ID-1 has been shown to be required for G1 progression, and its constitutive expression inhibited the lineage commitment and differentiation in B cells[21,22]. Second, in NHEK, among the down-regulated genes is G1 to S phase transition 1 (GSPT1), a homolog of the yeast gene GST1, which is essential for transition from G1 to S phase in the cell cycle[23,24].

### Late Response

From 12 to 24 h after NAC treatment, progressively more genes are affected in both cell types. Cluster analysis revealed tightly linked genes with multiple appearances, thus potentially more strongly implicated in the differentiation process. As a testimony to the lineage-specific differentiation programs, only very few genes were identified as being similarly regulated in both cell types. These included ID-1, Aquaporin 3 (AQP3), ErbB3, Cox-2, and HMG-box transcription factor 1 (HBP1). This last finding fits previous reports[25], where HBP1 negatively regulates Wnt/β-catenin, thus inhibiting proliferation and suggesting a tumor-suppressor function. Still in both cell lines, down-regulation of BMP-2 and of the TGF-β family member BMP-4 correlates well with the repressed ID-1 expression, since both BMP-2 and -4 up-regulate ID-1. Finally, and in accordance with immunolabeling data (see below), increased levels of E-cadherin were found in both Caco-2 and NHEK. In Caco-2, the differentiation-related gene NDRG1, which is expressed during differentiation and down-regulated in colorectal neoplasms[26], was up-regulated. In NHEK, up-regulated markers of differentiation include keratin 1 (KRT1), small proline-rich protein 2C (SPRR2C), and small proline-rich protein 4 (SPRR4). Keratins are the major structural proteins of differentiated keratinocytes; the SPRR gene family encoding precursor proteins to the cornified envelope is located on human chromosome 1q21, a region known as the epidermal differentiation complex, since expression of these genes is seen in maturing epidermal cells[27].

In agreement with cytometric analysis[14], it should be noted that apoptosis does not appear to act as a regulating mechanism, since only a very small proportion of apoptotic genes are affected in either direction (two to 12 genes out of 317). Actually, the HSP27 gene was up-regulated both in NHEK and in Caco-2[13,14]. The HSP27 gene product is reported to prevent apoptosis associated with growth arrest[28].

Hence, although induced by the same mechanism (NAC) and yielding the same end point of growth inhibition and differentiation, the processes in NHEK and Caco-2 are, on the whole, different.

## DEACTIVATION AND RELOCALIZATION OF C-SRC TYROSINE KINASE BY NAC

Tyrosine phosphatases are among the proteins whose function can be regulated through redox transition in sensitive Cys residues; their inhibition by oxidation and activation by reduction has been clarified in detail[29]. The case of the nonreceptor tyrosine kinase c-Src was lately brought into focus. This kinase is the product of the first discovered and, possibly, most investigated proto-oncogene, whose multiple functions and regulation mechanisms are still far from being fully elucidated[30]. Overexpression of the c-SRC protein and an increase in specific activity has been observed in several malignant conditions, in which it plays several roles in the transduction of signals relevant to the switch between cell growth and differentiation, delivery of oncogenes to the nucleus, cell motility, to mention just a few significant examples. In particular, active c-Src can disassemble cell-cell junctions by phosphorylating E-cadherin and β-catenin, and as a consequence, this last protein is delivered to the nucleus, where it exerts an oncogenic effect. Colorectal cancer shows a progressive increase in c-Src activity as the tumor stage advances, and elevated protein levels and/or catalytic activity of c-Src have been detected in lung, skin, colon, breast, ovarian, endometrial, head and neck, and prostate malignancies[31,32,33]. Because of its central role in cancer biology, c-Src is seen as an important target for therapeutic interventions and major drug-discovery efforts are focused on the development of specific inhibitors[34,35].

The first discovered regulatory switch for c-Src activity is primed by selective phosphorylation of alternative tyrosine residues. Activation follows the autophosphorylation of tyrosine Tyr419 in the SH1 domain, leading to an open, substrate-accessible conformation. Inactivation, on the other hand, and a “closed” conformation follow the phosphorylation of Tyr530 in the regulatory C-terminal domain by the carboxy terminal Src kinase (Csk)[30].

The subcellular localization of c-Src is also functionally relevant, being the active enzyme anchored to the plasma membrane, where it cooperates in signal transduction of ligand-activated cell-surface receptors. The inactive form, instead, is found in cytoplasmic compartments identified as late endosomes, generating a “gradient of Src activation”, shifting from the inactive form in perinuclear compartments to the active form at the membrane[36,37,38].

A regulatory mechanism based on a thiol oxidation has been more recently proposed. Oxidation of Cys residues by nitric oxide or peroxynitrite was shown to produce an increase of Tyr419 autophosphorylation[33,39,40].

When NAC was tested on c-Src preactivated with hydrogen peroxide, a clear deactivating effect was detected, which completely reverted the oxidation-driven activation[15]. The typical reductant dithiotreitol (DTT) had a similar effect, as did GSH, although less efficiently (Fig. 1A). Under these conditions, Tyr419 phosphorylation was decreased by 24 ± 6%.

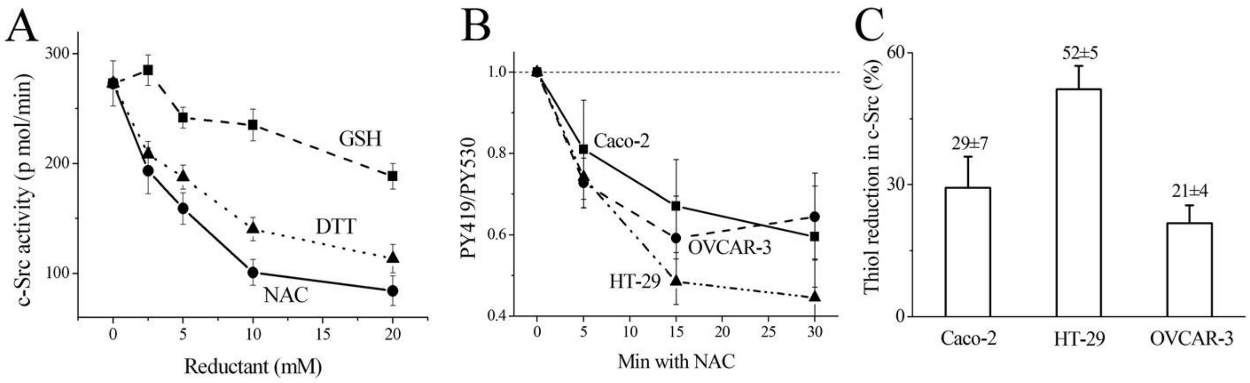

**FIGURE 1**. NAC reverted c-Src activation. (A) The activity of purified c-Src was analyzed as the phosphorylation rate of a specific substrate peptide and was found reduced in a dose-dependent fashion by NAC, DTT, and GSH. (B) The phosphorylation of Tyr419 and Tyr530 in c-Src was measured in cells incubated in the presence of NAC by Western Blot. Tyr530 phosphorylation was either unaffected or slightly increased, so that when reporting the Tyr419/Tyr530 ratio, the effect of NAC is quite clear. In all cells, the PTyr419 decreased up to approximately 30% after 30 min in the presence of NAC. (C) Increased accessibility to BIAM labeling, a selective probe of reduced thiols in cysteine, in c-Src immunoprecipitated from the three cell lines after 15 min from NAC treatment. A and C are redrawn from Krasnowska et al.[15].

NAC can also decrease c-Src Tyr419 phosphorylation in cancer cells. As examples of cancer cells where c-Src is activated, we used the colon and the ovary carcinoma cells Caco-2 and OVCAR-3, for which we had already obtained evidence of reduced c-Src activity following NAC supplementation[14]. We also used the colon carcinoma cell line HT29, due to its abundance in activated c-Src. In all lines, the phosphorylation of Tyr419 progressively decreased, up to approximately 30% after 30 min in the presence of NAC. Tyr530 phosphorylation, instead, was either unaffected or slightly increased (Fig. 1B).

This decrease in c-Src activity is actually dependent on the redox status of sensitive Cys in the kinase, a higher labeling of reduced thiols in immunoprecipitated c-Src being monitored after NAC treatment (Fig. 1C).

In agreement with the finding that activation of c-Src is associated with the transfer of this kinase from the perinuclear region of the cell cytoplasm to the plasma membrane inner surface[36,37,38], conversely, NAC treatment shifted c-Src out of the membrane towards organelles located around the nucleus then identified as acidic endosomes[15]. In Fig. 2, we report an example of such a relocation in OVCAR-3 cells, showing c-Src, with phosphorylated Tyr419, shifted from the cell membrane to endosomes. Clearly this relocation occurs independently on the active/inactive state of c-Src that, after 15 min from NAC treatment, is still mainly active (Fig. 1).

In the emerging picture, redox transitions of sensitive cysteines in c-Src activate a conformational shift, in turn affecting the kinase activity. The demonstration that this kinase can also be deactivated by thiol-reducing agents brings into focus the new, and appealing, option of targeting this mechanism so as to inhibit the kinase.

## PROLIFERATIVE AND MORPHOLOGICAL CHANGES IN NAC-TREATED NORMAL AND CANCER CELLS

Inhibition of cell proliferation after *in vitro* treatment with NAC was observed in several cases, with the relative increase of cells in the G1 phase[10,41,42]. In our experiments, we determined that both normal and cancer cell proliferation could be inhibited by NAC, although the required effective dose was

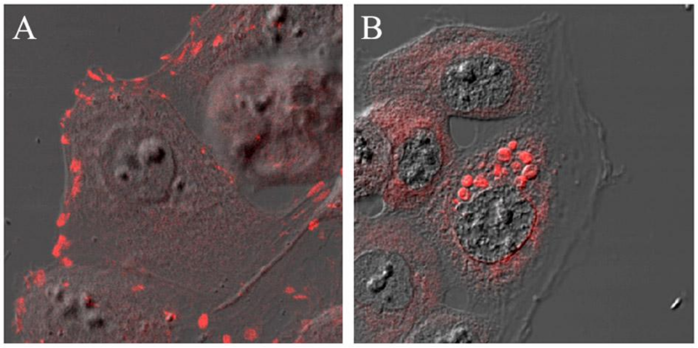

**FIGURE 2.** NAC treatment drives c-Src, mostly in the active Tyr419-phosphorylated form, into acidic endosomes. Confocal images of c-Src antiphosphoTyr419 (red) merged with phase contrast images, in control (A) and NAC-treated (15 min) (B) OVCAR-3 cells. Frame: 77 µm. Redrawn from Krasnowska et al.[15].

different. While 2 mM NAC was sufficient to block the proliferation of normal keratinocytes completely, colon carcinoma cells required 10 mM NAC and with this last concentration, only half of the ovary carcinoma cell population did not proliferate.

In agreement with the generally observed inhibition of apoptosis due to NAC[41], cytometric measurements showed that the decreased proliferation in NAC-treated cells was not dependent on apoptosis. Still, in agreement with the gene expression analysis and with the inhibition of the key tyrosine kinase c-Src, we detected the formation of a differentiated morphology in NAC-treated cells, showing that the nonproliferating cells definitely proceed towards a differentiation pathway. In normal keratinocytes, cornified envelope appeared in 3 days, with a tenfold acceleration relative to untreated cells, and in carcinoma cells, various signatures of a terminal differentiation also appeared in 3 days.

On the whole, as reported in detail[14], at a morphological level, in NAC-treated cells, we can observe: (1) a regular polygonal morphology, as opposed to the mesenchymal morphology of proliferating cells (Fig. 3); (2) a *de novo* formation of the brush-border microvillar structure in colon carcinoma cells and of the cornified envelope in keratinocytes (Figs. 3 and 4); (3) the basal relocation of the nucleus in colon carcinoma cells together with the formation of a robust apical vesicular apparatus (Fig. 4); (4) an overall rearrangement of the cytoskeleton to fulfill the requirements of newly formed structures; (5) the increase of cell-cell and cell-substratum junctions (Fig. 5); and, finally, (6) the disappearance of the oncogenic β-catenin from the nucleus and its confinement at the cell-cell junctions (Fig. 5). Taken together, the relocation of β-catenin at the level of junctions and the increased concentration of E-cadherin[14] represent both markers and inducers of differentiation[43,44], indicating the establishment of a positive feedback in the differentiation process. The increase in cell-cell and cell-substratum adhesions identifies a more adhesive and less motile phenotype. Also, an increased cell-substratum adhesion has been indicated as a differentiation marker.

## CONCLUSION

The number of reports in the literature concerning NAC is so wide that one can doubt the ability to review them critically in an average lifetime. Nevertheless, it is easy to report that most of them refer to NAC generically seen as a free-radical scavenger antioxidant. However, based on the increasing knowledge on thiol redox transitions in cell signaling, we foresee that NAC interferes with the transduction of proliferation/differentiation signaling by a nonradical mechanism. From the chemistry of redox signaling, disulfide redox switches emerge as the most likely targets of NAC activity. NAC treatment in normal and cancer cells involves a massive rearrangement of gene expression profile, accompanied with biochemical

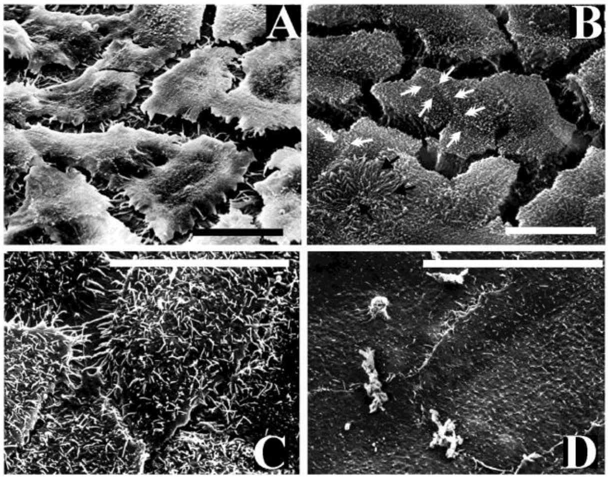

**FIGURE 3**. Effect of NAC treatment on cell morphology. Scanning electron micrographs of colon carcinoma, Caco-2 cells (A,B) and of normal primary keratinocytes, NHEK (C,D), untreated controls (A,C) and NAC-treated (B,D). Proliferating Caco-2 cells (A) display an irregular morphology with scarce microvillous structures and large intercellular space. After 72 h with 2 mM NAC, Caco-2 cells (B) had the morphology of end-stage differentiated cells, regularly polygonal and about three times thicker than controls, with a relevant number of brush-border microvilli at the cell surface (black arrows) and a dramatically reduced intercellular space (white arrows). Bars: 20 µm. NHEK-untreated controls (C) show a heterogeneous polygonal morphology, with a villous surface and relatively broad intercellular space. After 72 h with 2 mM NAC, cells grow flat in a thin monolayer with a regular polygonal morphology and a smooth surface, with loss of the fine microvillous structure, with dramatically reduced intercellular space. Bars: 25 µm. Redrawn from Gustafsson et al.[13] and Parasassi et al.[14].

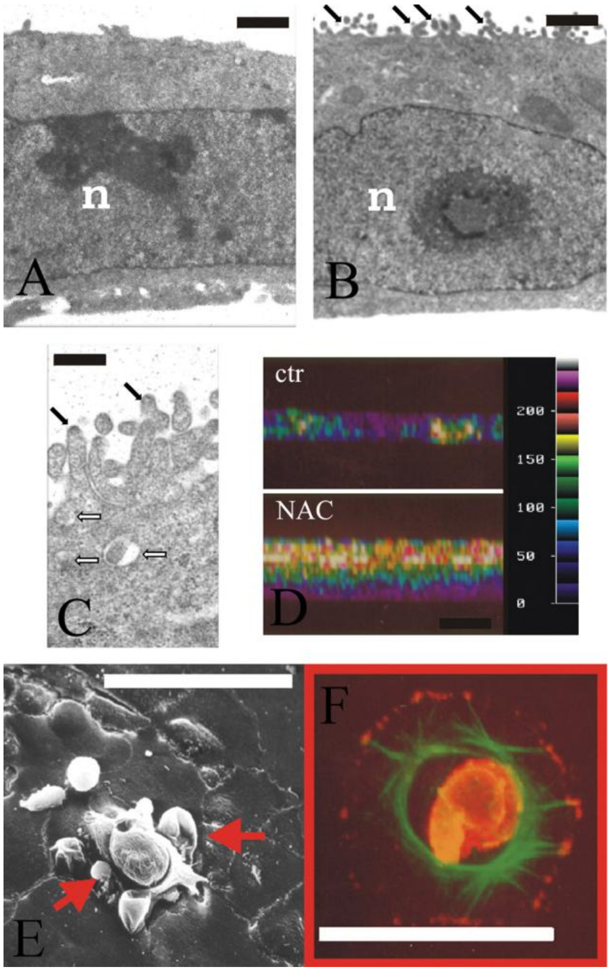

**FIGURE 4.** Differentiation of cells treated with NAC. Transmission electron micrographs of control (A) and of NAC-treated Caco-2 cells (B,C). Arrows in (B) and (C) point to some brush-border microvilli; open arrows in (C) point to secretory vesicles. (D) Confocal fluorescence micrographs of intracellular secretory vesicles in Caco-2 cells – Golgi apparatus and endoplasmic reticulum – labeled by TRITC-conjugated wheat germ agglutinin. Longitudinal sectioning along the x/z plane of control (top) and NAC-treated (bottom) cells allows a clear visualization of the apical distribution and of the increased amount of secretory vesicles after NAC treatment, as well as a relevant increase in thickness, indicating a differentiated function and morphology. Intensity color scale on the right. (E) Scanning electron micrograph of initial stage of cornified envelope in NHEK, with an enucleated cell (arrows). (F) Confocal fluorescence micrograph in NHEK of an actin-vinculin (green and red, respectively) double-stained cornified cell showing that actin microfilaments participate in the enucleation process. Bars: 1 µm in (A,B); 300 nm in (C); 10 µm in (D); 100 µm in (E); 25 µm in (F). Redrawn from Parasassi et al.[14].

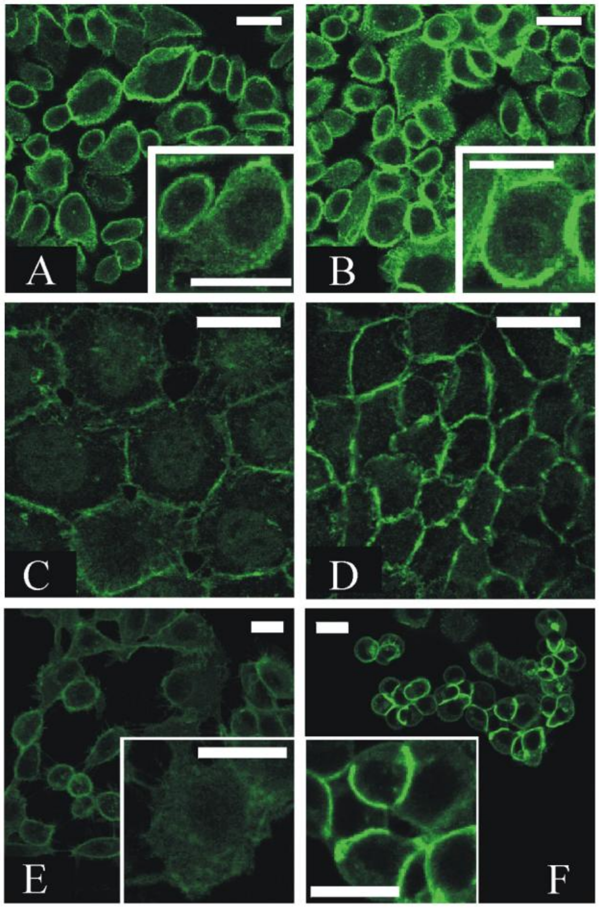

**FIGURE 5**. Effect of NAC treatment on β-catenin increase and relocation. β-Catenin labeling in NHEK (A,B), Caco-2 (C,D) with details at higher magnification in the lower insets, and OVCAR-3 (E,F) after 3 days of growth. Control cells in the left column (A,C,E) and after 3 days from NAC treatment in the right column (B,D,F). NHEK grown in the presence of 2 mM NAC (B) show an increased number of intercellular junctions, with bound β-catenin. In Caco-2 cells grown in the presence of 5 mM NAC (D), the intracellular localization of β-catenin was also greatly affected, with redistribution from the nucleus to adherens junctions. From the image analysis, the fluorescence intensity increased of a factor of 7 relative to controls. A morphological change can be also observed, with smaller and more polygonal cells after NAC treatment. After 3 days from a 5 mM NAC treatment, OVCAR-3 cells (F) show the β-catenin disappearance from the nucleus, while it increases at the level of cell-cell junctions. From the image analysis, β-catenin fluorescence intensity at the junctions increases a factor of 30 with respect to controls. Morphological changes can be observed, from large and flat to smaller and regularly polygonal. Bars: 25 µm. Redrawn from Parasassi et al.[14].

and morphological changes, all converging towards terminal differentiation. This opens attractive perspectives, both for the use of this compound in the study of redox signaling and also in complementary interventions for cancer treatment.

## ACKNOWLEDGMENTS

This study was supported in part (TP, GC, EKK, TL, EP) by Iasomai AB, Stockholm, Sweden.
